# Effects of language stimulation on cognition of institutionalized aged people: a preliminary case series study

**DOI:** 10.1590/1980-57642021dn15-010015

**Published:** 2021

**Authors:** Ruttienya Dias Braga da Cunha, Tayane Frez Pacheco, Simone dos Santos Barreto

**Affiliations:** 1 Universidade Federal Fluminense – Nova Friburgo, RJ, Brazil.; 2 Instituto Israelita de Ensino e Pesquisa Albert Einstein – Rio de Janeiro, RJ, Brazil.; 3 Department of Specific Training in Speech-Language Pathology, Instituto de Saúde de Nova Friburgo, Universidade Federal Fluminense – Nova Friburgo, RJ, Brazil.

**Keywords:** language, cognition, homes for the aged, language therapy, treatment outcome, linguagem, cognição, instituição de longa permanência para idosos, terapia da linguagem, resultado do tratamento

## Abstract

Cognitive stimulation programs for institutionalized elderly people show positive results, however few studies have investigated the effectiveness of language stimulation programs for the health of this population.

**Objectives:**

To characterize the cognitive-linguistic profile of institutionalized elderly and to compare their performance before and after a language stimulation program (LSP).

**Methods:**

An exploratory case series study was conducted with nine residents of a Home for the Aged. Elderly people aged 60 or over, of both sexes, without neurological or neuropsychiatric diseases, communication disorders, intellectual impairment or severe visual or hearing impairment were included. The participants were submitted to an initial assessment through the Montreal Cognitive Assessment (MoCA) and Montreal Toulouse Battery Language Assessment – Brazil to characterize the cognitive-linguistic profile of the studied group. Five elderly were selected to participate in the LSP, of wich only two participated effectively in the program, but all were reassessed after the program was completed.

**Results:**

on the initial assessment, of the nine participants, only one had adequate cognitive performance and all presented changes in macro and/or microlinguistics aspects of oral discourse, with oral comprehension preserved. On the reassessment carried out with five participants, only two participants who adhered effectively to the program obtained improvements in MoCa scores. In regarding language, three participants performed better in the oral emission measures. The performance of the participants in oral comprehension remained or declined.

**Conclusion:**

The speech-language therapy intervention through a LSP contributes to improving the cognitive-linguistic performance of institutionalized elderly.

## RESUMO.

Programas de estimulação cognitiva voltados para idosos institucionalizados mostram resultados positivos, contudo poucos estudos investigaram a eficácia de programas de estimulação da linguagem para a saúde dessa população.

**Objetivos:**

Caracterizar o perfil cognitivo-linguístico de idosos institucionalizados e comparar o desempenho antes e após um programa de estimulação de linguagem (PEL).

**Métodos:**

Foi realizado um estudo exploratório do tipo série de casos, com nove residentes de instituição de longa permanência para idosos (ILPI). Incluíram-se idosos com 60 anos ou mais, de ambos os sexos, sem doenças neurológicas ou neuropsiquiátricas, distúrbios da comunicação, deficiência intelectual, ou deficiência visual ou auditiva grave. Os participantes foram submetidos a uma avaliação inicial, com a Montreal Cognitive Assessment (MoCa) e a Bateria Montreal Toulouse de Avaliação da Linguagem – Brasil, para caracterizar o perfil linguístico-cognitivo do grupo estudado. Foram selecionados cinco idosos para participar do PEL, dentre os quais apenas dois participaram efetivamente do programa, porém todos foram reavaliados imediatamente após a finalização do programa.

**Resultados:**

Na avaliação inicial, dos nove participantes, apenas um participante apresentou desempenho cognitivo adequado, e todos apresentam alteração de aspectos macro e/ou microlinguísticos do discurso oral, com compreensão preservada. Na reavaliação realizada com cinco participantes, apenas os dois que aderiram efetivamente ao programa obtiveram melhoras no escores do MoCa. Em relação à linguagem, três participantes apresentaram melhor desempenho em emissão oral. O desempenho dos participantes em compreensão oral manteve-se ou declinou.

**Conclusão:**

A intervenção fonoaudiológica por meio de um PEL contribui para a melhora do desempenho cognitivo de idosos institucionalizados.

## INTRODUCTION

In the year 2050, the aged population in Brazil will be some 3.7 times greater than in 2000, standing at around 49 million.^1^ This rise is due, among other factors, to the increased survival of the older population, as a result of improvements in health care for this group.^2^ Thus, the need for greater vigilance among this age group is clear, given the many challenges in providing this rapidly growing population with the chance of a healthy active old age.^3^ One of these challenges is the burden families face in caring for older people in their own homes,^4^ leading to an increased demand for homes for the aged.

Institutionalization can lead to limitations in the social functioning of elderly residents who, in most cases, cease to perform their usual daily tasks due to the dynamics of the facility, causing loss of autonomy and independence.^5^ Preserving cognition in institutionalized aged individuals is paramount to ensure that residents can carry out their activities and maintain the ability to perform self-care.^6^ Another factor which should be taken into account is communication, since this function can be impaired due to cognitive decline,^7^ with repercussions on social participation and individual behavior.^8^ Therefore, communication must not be overlooked during assessment and follow-up of institutionalized elderly.

Acquired language disorders, such as aphasias and linguistic-cognitive disorders, are common in this population, resulting from strokes and dementia. Language skills in these cases may be more compromised when aged people are institutionalized.^9^ Therefore, the early stimulation of these skills is indicated for the maintenance of social interactions and the quality of life of these elderly.^8^

The benefits promoted by cognitive stimulation programs for elderly residents of homes for the aged has already been described in a national study.^9^ In this study, the cognitive stimulation promoted benefits among institutionalized elderly, despite the low educational level of the group and the limitations in intervention accessibility and performance.^10^

Regarding language abilities, few studies have investigated the effectiveness of language stimulation programs for the health of this population.^11^ ^–^ ^13^ Favorable results for greater social interaction were pointed in a qualitative study with 10 neurologically healthy aged people, exposed to linguistic-discursive activities in 16 group meetings held at a home for the aged.^11^ Positive effects of interventions implemented were also observed in two single-case studies of aged people with dementia residing at a home for the aged.^12^ ^,^ ^13^ However, the associations between language and cognitive skills were not investigated in these studies.

The present study sought to contribute with new evidence by investigating the effects of language stimulation on the cognition of institutionalized aged people. Therefore, this study aimed to characterize the cognitive-linguistic profile of the institutionalized aged and to compare performance before and after a Language Stimulation Program (LSP).

## METHODS

An exploratory case series study approved by the Research Ethics Committee of the lead institution (permit No. 3.281.461) was conducted. The study involved aged residents of a home for the aged housing 66 individuals, located in a city of Rio de Janeiro State. All participants and/or their legal guardians consented and/or agreed to take part in the study, conducted in 2019.

From this population, a sample of 11 participants was recruited, aged 60 years old or older, of both genders, with no history of neurological or neuropsychiatric disease and/or no current or previous diagnosis of communication disorders. The remaining residents were excluded for presenting neuropsychiatric conditions, including moderate or severe dementia, or intellectual disability, blindness, or non-corrected poor vision and/or deafness, which precluded standard assessment of cognition and/or language. Of the 11 residents who met the inclusion criteria, 9 agreed to take part in the study and underwent the initial assessment to characterize cognitive-linguistic profile.

The second stage involved the selection of participants for inclusion in the LSP. They were selected from the 9 participants in the first stage of the research, in which the linguistic-cognitive profile of the group was investigated. For eligibility, participants’ performance had to lie within the normal range in one of the language subtests applied. After appliying the initial assessment, a sample of 6 individuals was included based on performance, although 1 resident subsequently died. None of the participants met the diagnostic criteria for neurogenic language disorders. Thus, a total of 5 aged individuals completed the final assessment, entailing application of the same instruments used in the first assessment. However, only 2 participants actually joined the program, often with sessions above 90%. Data on the other participants who did not join, that is, who participated in some sessions or none of them, were analyzed for comparison purposes.

Participants were assessed pre and post-LSP by applying two instruments, validated and standardized for use in aged Brazilian Portuguese speakers, namely: the Montreal Cognitive Assessment (MoCa), a screening test to differentiate patients with mild cognitive impairment (MCI) from aged people with normal cognitive aging,^14^ and the Montreal Toulouse Language Assessment Battery – Brazil (MTL-BR), used for screening for language disorders.^15^ In the present study, the MTL-BR subtests Oral narrative discourse and Oral text comprehension were used to analyze participants’ expressive and receptive language aspects, respectively.

Each brief evaluation (assessment or reassessment) was performed through one or two weekly sessions lasting up to 60 minutes each, conducted at the home for the aged. At each assessment, the MoCa was applied followed by the subtests of the MTL-BR Battery, as per standardized instructions, and answers were scored using criteria based on normative data for each test.^14^ ^,^ ^15^

The LSP is designed to promote the communication of institutionalized aged people, thereby improving their cognitive status and helping them maintain cognitive and linguistic abilities. The program was implemented in groups for 11 weeks, entailing 13 intervention sessions of 50 minutes each, whose therapeutic goals were: to optimize communicative practices among residents; and optimize both expressive and receptive aspects of spoken language. An overview of the program is presented in Chart 1, including specific goals and therapeutic strategies covered in each session.

Performance of the participants in the LSP was recorded at each session by making individualized notes and audio and/or video recordings, when applicable, to observe aspects related to auditory comprehension and oral expression of each participant during the group sessions. In addition, clinical evolution of participants from initial assessment to reassessment was tracked by collecting data from medical records. The following data were collected: medications administered, exams performed, behavior, health status and family assistance, encompassing aspects which might influence the results of this study.

Statistical analysis:

In view of the exploratory nature of the present study and the sample size, the data obtained were analyzed using descriptive statistics, including analysis of the distribution of relative frequency for categorical variables, and median, minimum, and maximum values for continuous variables. The independent variable analyzed was the adherence to the program. Dependent variables were: the total score on the MoCA test and the measures in the language subtests (number os words, number of information units (IU), number of scenes and score on the oral comprehension test). Pre- and post-LSP results were compared by calculating the difference between the scores attained at the two assessments, with results plotted in line graphics and bar charts.

## RESULTS

At the time of recruiting participants for the study, only 16.7% of the aged residents of homes for the aged had no diagnosed neuropsychiatric diseases or visual and/or hearing loss in their medical charts. Of the sample of 9 participants who agreed to take part in the study, most were male (66.6%) and age ranged from 64 to 93 years (median=1 years). Regarding educational level, 88.9% had ≤8 years of formal schooling. Time institutionalized ranged from 7 to 144 months.

On the initial assessment, MoCA scores ranged from 10 to 27 (median=16). Only one participant had a score within the normal expected range (≥26). Regarding linguistic performance, in the oral expression subtest of the MTL-BR Battery (Oral narrative discourse), participants produced discourse of 7 to 144 words (median=53) and only one participant a score below normal for this measure.^15^ Median scores in the other measures of this subtest were: 2 points for IU, range 0–8 points, and 0 points for scene elements (minimum: 0; maximum: 3), suggesting impairment of micro/macrolinguistic aspects of oral discourse.^15^ Only 33.3% of the group produced coherent and cohesive narratives. In the auditory comprehension subtest of the MTL-BR Battery (oral text comprehension), participants scored 2–8 points, median of 5 points and, thus, performance was within normal parameters.^15^ However, the performance of 3 participants was below normal. Results of these assessments by participants in each MoCa task or MTL-BR subtest item are given in Table 1.

**Table 1:** Cognitive and linguistic performance of institutionalized elderly.

Tests		Scores of participant								
		P1	P2	P3+	P4+	P5	P6§	P7	P8	P9
MoCa										
	Executive functions (1/5)	0	0	0	0	0	4	0	0	0
	Naming (4/3)	3	4	3	4	2	3	3	2	4
	Memory/ delayed recall (5/5)	1	0	0	0	0	3	2	1	0
	Attention (3/3)	0	3	1	0	0	3	1	1	0
	Language/ fluency (2/3)	0	1	1	1	1	2	1	0	1
	Abstraction (3/2)	2	2	1	1	1	2	1	2	1
	Orientation (6/6)	4	6	6	6	4	6	5	4	4
	Calculation (3/3)	1	3	3	2	1	3	0	0	3
	Visual perception (3/0)	0	3	3	1	0	---	0	0	2
	Extra point (schooling)	1	1	1	1	1	1	1	1	1
	Total score (30)	12	23	19	16	10	27*	14	11	16
MTL-Oral narrative										
	Number of words	17*	93*	100*	37*	53*	144*	21*	7	55*
	Information units (10)	1	6*	4*	1	1	8*	2	0	2
	Scenes (3)	0	2*	2*	0	0	3*	0	0	0
	Cohesion (1)	1	1	1	1	0	1	0	0	0
	Coherence (1)	0	1	1	0	0	1	1	0	0
MTL-Oral text comprehension										
	Total (9)	7*	5*	7*	6*	2	8*	5*	3	2

MoCa: Montreal Cognitive Assessment; MTL: Montreal Toulouse Language Battery;

* Performance above the cut-off score;

+ elderly people aged 80 years or older;

§ scores vary due to applied version.

Sociodemographic data of the 5 participants who participated in the LSP and completed the final assessment are presented in Chart 2. They had different levels of intervention adherence, from 92 to 0% of attendance to the LSP sessions (Chart 2). Thus, they were grouped into: subgroup 1 (P1 and P7), which effectively joined the program; subgroup 2 (P3 and P6), which participated in less than 1/3 of the sessions; and subgroup 3, composed only of P2, who did not participate in the program.

**Figure 1:**
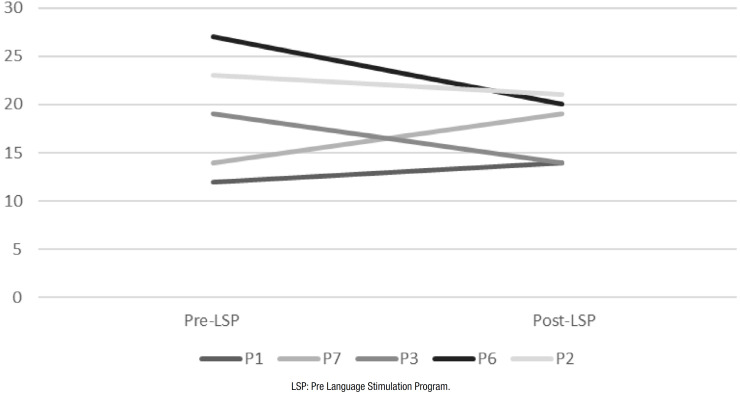
Performance in the Montreal Cognitive Assessment test on assessment and reassessment.

Total MoCa scores for each participant in the initial assessment and reassessment are depicted in Figure 1. Results show that the scores of participants in subgroup 1 improved in the reassessment. Participants’ performance in the MoCa test for each task, together with the difference between assessment and reassessment, are given in Table 2.

**Table 2:** Performance in the Montreal Cognitive Assessment test tasks for assessment and reassessment.

MoCa tasks	Performance														
	P1			P7			P3+			P6§			P2		
	As||	Re¶	Dif**	As	Re	Dif	As	Re	Dif	As	Re	Dif	As	Re	Dif
Executive functions (1/5)	0	0	0	0	0	0	0	0	0	4	4	0	0	0	0
Naming (4/3)	3	4	-1	3	3	0	3	4	-1	3	3	0	4	4	0
Memory/delayed recall (5/5)	1	0	1	2	2	0	0	0	0	3	2	1	0	1	-1
Attention (3/3)	0	0	0	1	3	-2	1	0	1	3	3	0	3	2	1
Language/fluency (2/3)	0	0	0	1	1	0	1	0	1	2	1	1	1	1	0
Abstraction (3/2)	2	2	0	1	2	-1	1	1	0	2	0	2	2	1	1
Orientation (6/6)	4	5	-1	5	4	1	6	4	2	6	5	1	6	6	0
Calculation (3/3)	1	2	-1	0	1	-1	3	3	0	3	1	2	3	3	0
Visual perception (3/0)	0	0	0	0	2	-2	3	2	1	---	---	0	3	3	0
Total	12	14	-2	14	19	-5	19	15	4	27*	20	7	23	22	1

MoCa: Montreal Cognitive Assessment;

* Performance above the cut-off score;

+ elderly people aged 80 years;

§ sores vary due to applied version;

|| assessment;

¶ reassessment,

** difference.

**Figure 2:**
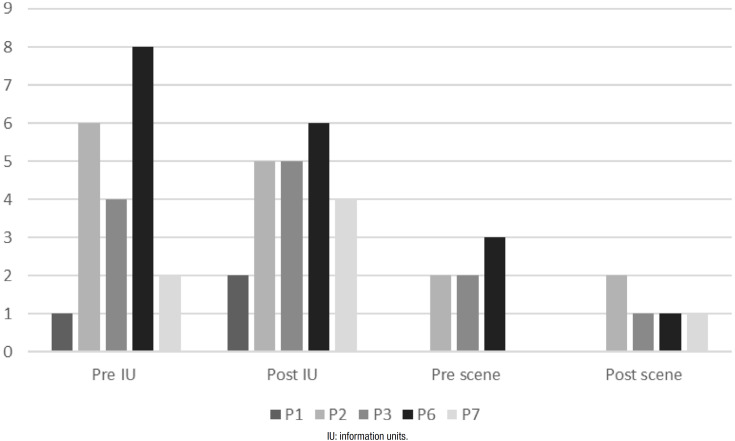
Performance in the Oral narrative subtest of the Montreal Toulouse Language Battery on assessment and reassessment.

Participants’ performance for the measures of oral expression (IU and scenes) of the MTL-BR Battery in the two assessments is depicted in Figure 2. Results show that 3 participants improved IU scores (subgroups 1 and 2), whereas only 1 improved scores in the MTL scenes (subgroup 1).

**Figure 3:**
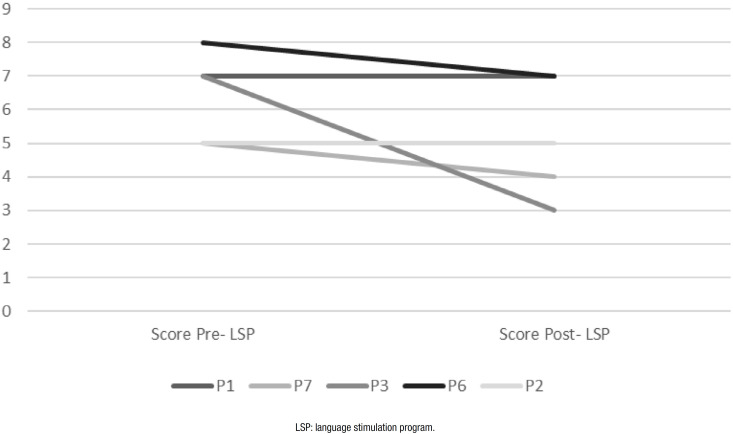
Performance of the participants in the Oral text comprehension of the Montreal Toulouse Language Battery on the assessment and reassessment.

The total scores for the oral text comprehension test at assessment and reassessment were compared (Figure 3). Performance in oral text comprehension either declined (subgroups 1 and 2) or remained stable (subgroups 1 and 3) for all participants.

The scores of the participants in the oral narrative discourse and in oral text comprehension for the MTL-BR Battery subtests at assessment and reassessment, along with the difference between them for each measure, are given in Table 3.

**Table 3:** Performance on the Montreal Toulouse Language Assessment-Brazil Battery subtests for assessment and reassessment.

MTL-Brazil subtests		Performance														
		P1			P7			P3			P6			P2		
		As+	Re§	Dif||	As	Re	Dif	As	Re	Dif	As	Re	Dif	As	Re	Dif
Oral narrative																
	Number of words	17*	45*	-28	21*	37*	-16	100*	102*	-2	144*	47*	-97	93*	99*	-6
	Information units	1	2	-1	2	4*	-2	4*	5*	-1	8*	6*	2	6*	5*	1
	Scenes	0	0	0	0	1*	-1	2*	1*	1	3*	1*	1	2*	2*	0
	Cohesion	1	1	0	0	0	0	1	1	0	1	1	0	1	1	0
	Coherence	0	0	0	1	1	0	1	1	0	1	1	0	1	1	0
Oral text comprehension																
	Total	7*	7*	0	5*	4*	1	7*	3	4	8*	7*	1	5*	5*	0

MTL: Montreal Toulouse Language Battery;

* Performance above the cut-off score;

+ assessment;

§ reassessment;

|| difference.

A summary of the most relevant findings for participants’ clinical progression between assessment and reassessment by subgroup is given in Chart 3.

## DISCUSSION

Most of the participants who underwent the initial assessment through MoCa performed less than expected (88.9%), despite having no neuropsychiatric diagnosis recorded in their medical charts. The results of a previous Brazilian study assessing the performance of community-dwelling older adults revealed conditions related to probable cognitive decline/dementia in 26% of the studied sample (n=99),^16^ suggesting that the number of aged individuals with these impairments in homes for the aged could be considerable. According to Mello et al.,^17^ institutionalization is a factor that can contribute to a decline in cognitive performance of aged residents. In their study, 39.3% of participants presented cognitive impairment.^17^

The current analysis of individual performance of each participant (Table 1) revealed that everyone committed errors involving more than one cognitive ability. Performance in tasks involving executive functions, delayed recall/memory, attention, and visuoperceptual ability was affected in a greater number of residents (4–8 participants), all of whom scored zero. Regarding the language/fluency and abstraction tasks, over 50% of participants scored half the total or less, indicating a higher rate of impairment of these abilities.

For the group's language performance, this ability was more preserved than cognitive performance. Of the language measures used, the group's performance was within the expected range in the oral text comprehension subtest, but lower for the oral expression of the discourse. In cases of MCI, there is a pattern of linguistic impairment involving more specifically the semantic level and emissive tasks, such as naming, verbal fluency and processing at the discursive level.^18^ A total of 33.3% of participants had normal performance in the oral expression and comprehension measures (Table 1).

Comparison of scores on the MoCa for the initial assessment and the reassessment (Figure 1), showed that the LSP promoted improvement in overall cognitive performance, as measured by the total test score. Only participants of subgroup 1 (P1 and P7) who fully adhered to the program had higher scores in the reassessment. As shown in Table 2, P1 improved in naming, orientation, and calculation tasks, but worsened in memory ones; whereas P7 improved performance in attention, abstraction, calculation, and visuospatial tasks, but declined in the orientation task. Tasks whose performance varied between assessments were not common in these two cases, except for the improvement in the calculation task, whose relationship with language skills is quite specific.^19^ Considering that a cognitive screening test was used, it is not possible to analyze the possible impact of linguistic stimulation on specific cognitive skills. Future research using cognitive assessment batteries may contribute to a better understanding of these possible associations.

Participants of subgroups 2 (P3 and P6) and 3 (P2) showed no improvement in the total score on reassessment. Participant P6, whose performance was normal in the initial assessment, had below average performance in reassessment. With regard to subgroup 2, clinical deterioration occurred during the course of the study, which may have contributed to the performance decline seen in these participants during reassessment, such as behavioral disorders and medication use (Chart 3).^20^ The performance of participant P2 in the MoCa also worsened, albeit to a lesser extent. Although this individual had no relevant clinical complications in the period, the subject displayed social engagement constraints, with limited social interaction practices, refusing to take part in activities at the home. Social isolation and institutionalization are risk factors for the development of cognitive dysfunctions and, therefore, the lower scores might be partially explained by these aspects. Thus, it is vital that professionals working in these environments devise strategies to enable communication and help users to better adapt to the changes brought about by institutionalization.^11^ ^,^ ^21^

The results of this preliminary study suggest that group language stimulation may help improve cognitive abilities in institutionalized aged people with impaired cognition and oral expression, exemplified by the cases analyzed. In the neurolinguistics field, human interactions and language are regarded by many scholars as a fundamental factor for the development of cognition.^8^ ^,^ ^11^ ^,^ ^22^

With regard to the possible effects of LSP on the linguistic performance of the participants, for oral expression abilities (Figure 2), IU scores increased in the two members of subgroup 1 (P1 and P7) and also in P3 from subgroup 2, but decreased in the other cases. Resident P7 attained the cut-off score for this measure, indicating no impairment in this aspect of the discourse after intervention. The declines seen in the other cases did not translate into impairment of this ability, *i.e*., their scores remained within normal limits on reassessment. Participant P3, whose involvement in the program was greater than that of P6, showed improvement in this task, indicating that a minimum number of sessions is required to yield positive results for oral discourse under the program. Case studies with institutionalized aged people with dementia indicated an improvement in lexical skills in the oral discourse after individualized LSP.^12^ ^,^ ^13^

The performance of users in terms of number of scenes revealed that the only participant who improved in the macrolinguistic aspect was part of subgroup 1, *i.e*., who adhered to the treatment (P7), whereas participant P1 did not improve in this parameter. Although the other participants failed to show performance improvements, their scores remained within normal limits, with no clinically relevant impairments detected. Additionally, no qualitative differences in discourse cohesion or coherence between the two assessments were observed. In the study by Marquete, with an institutionalized elderly woman with dementia, there was no change in this measure after the proposed LSP using the same test. However, when the evaluation took place through oral narrative discourse, based on a sequence of scenes, an improvement was observed in the production of macropropositions.^12^ Such evaluation strategies seem to be more sensitive according to the review carried out on language changes in MCI and dementia.^18^

For oral discourse comprehension, none of the participants exhibited improvements. However, performance remained within parameters of normality for this measure in reassessment, confirming no clinical decline in this ability among participants, except for P3 from subgroup 2, whose overall clinical status declined over the study period (Chart 3). In LSP applied to an institutionalized aged person with dementia mentioned above,^12^ who showed impaired oral comprehension, an improved performance was observed.

In terms of the LSP implementation, although designed as a group intervention, strategies were adapted to the specificities of each participant, such as pre-intervention cognitive profile, where this may have influenced the results of the subgroup which fully adhered to the program. Mapping the cognitive profile of the aged allows targeted treatment plans for personalized care, promoting strategies that produce greater satisfaction for residents of homes for the aged.^23^ Speech and language therapists, given their focus on communication, are best placed to implement therapeutic strategies that stimulate oral discourse abilities and communication exchange of residents with team members, relatives, and friends, creating a care environment that favors the cognitive health of these elderly. Such health promotion pratices contribute to the engagement of the aged in richer communicative interactions with their interlocutors and can contribute to the reduction of linguistic isolation and its consequences, including the cognition of this group.^24^ Additionally, this professional can contribute to the identification of changes in language skills in individuals at risk for MCI.^7^

The present study has several limitations. A limitation was the small sample size, due to the high level of morbidity found in the home for the aged, precluding the inclusion of many potential participants. Therefore, the therapeutic treatment plans of this study should be adapted to the situation of residents of each institution, determining which care practices meet their health needs, including end-of-life care. Another limitation involved the study type, preventing conclusions about the effectiveness of the program. The inclusion of more outcome measures related to language and cognition would also be necessary to assess the effects of the program. Further studies with an emphasis on clinical effectiveness should be conducted in the form of randomized clinical trials encompassing other previously investigated group therapy approaches.

The present study revealed that, in the initial assessment, all participants had evidence of impairment in more than one cognitive ability. In the group studied, language proved more preserved than other cognitive functions, particularly oral comprehension of discourse. The LSP promoted an improvement in cognitive performance, assessed by the total MoCa score, and oral language expression of those residents who adhered effectively to the group intervention. However, no positive effects on the participants’ oral comprehension abilities were evident. This finding highlights the need to adapt the program, particularly its oral comprehension strategies, so it can be used in future studies. Further studies will be necessary considering the preliminary nature of this study.
